# Thermodynamics of Water Displacement from Binding Sites and its Contributions to Supramolecular and Biomolecular Affinity

**DOI:** 10.1002/anie.202505713

**Published:** 2025-06-16

**Authors:** Jeffry Setiadi, Frank Biedermann, Werner M. Nau, Michael K. Gilson

**Affiliations:** ^1^ Skaggs School of Pharmacy and Pharmaceutical Sciences University of California San Diego 9255 Pharmacy Lane La Jolla California 92093 USA; ^2^ Institute of Nanotechnology (INT) Karlsruhe Institute of Technology (KIT) Hermann‐von‐Helmholtz Platz 1 76344 Eggenstein‐Leopoldshafen Germany; ^3^ School of Science Constructor University Campus Ring 1 28759 Bremen Germany

**Keywords:** Cucurbiturils, Host‐guest complexes, Hydrophobicity, Macrocycles, MD simulations

## Abstract

The role of water displacement in noncovalent binding has been debated in the fields of supramolecular chemistry and drug design. We use molecular dynamics simulations of idealized host‐guest systems to address the long‐standing controversy of whether water is merely a bystander or an actual driver of noncovalent binding in aqueous solution. To isolate hydration effects, we consider a pseudo‐hard‐sphere guest binding to a series of cucurbit[8]uril‐based macrocyclic host models whose energetic interactions with water vary widely. The computed free energy cost of displacing water from binding sites ranges from 0 to +37 kcal mol^−1^, strongly influencing binding affinities. However, neither water density nor excess chemical potential reliably indicates the thermodynamic favorability of cavity water. These results support the concept that “unfavorable” binding site water contributes to high‐affinity binding and resolve the paradox of stable but thermodynamically unfavorable cavity water. This work provides insights into the nature of the hydrophobic effect in molecular recognition and offers a framework for understanding the role of water in binding across various host‐guest and protein‐ligand systems.

## Graphical abstract

The affinity between a guest and a macrocyclic host depends strongly on the water encapsulated inside the host cavity, with free‐energy variations on the order of 20 kcal mol^−1^ being possible even in the absence of direct host‐guest interactions. This conclusion–which contrasts alternative views–can be drawn from molecular dynamics simulations for the binding of a guest with pseudo‐hard‐sphere potential and cucurbit[8]uril host models with varying polarity and polarizability.

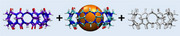

## Introduction

Life is based on water as a medium, and almost all key biological processes, for example, the self‐assembly of lipid bilayers, the folding of proteins, ligand‐receptor docking, enzyme‐substrate binding, and nucleic acid hybridization, involve the displacement of water from molecular surfaces. It is therefore intriguing that the degree to which–or indeed whether at all–the displacement of water molecules from binding sites and interaction surfaces plays an active driving role in these assembly processes has remained a matter of vivid debate. Even turning to the simplest model systems for these phenomena, namely macrocyclic hosts that bind hydrophobic guest molecules, has not resolved this question, but has, instead, escalated the controversy.

Indisputably, the binding of a guest molecule within the cavity of a macrocyclic host in water displaces, or releases, water from the binding site. Bender and Connors suggested early that the water in the nonpolar binding sites of cyclodextrins was “enthalpy‐rich”, “high‐energy”, or “energy‐rich”,^[^ ^1^, ^2^, ^3^ ^]^ so that its displacement makes a favorable enthalpic contribution to the binding free energy. The idea that water in a nonpolar cavity exists in a high enthalpy state appeared to contradict the traditional idea that the hydrophobic effect is entropy‐driven,^[^ ^4^ ^]^ rather than enthalpy‐driven, so Diederich coined the term “non‐classical hydrophobic effect” for such cases.^[^ ^5^, ^6^ ^]^ Subsequent work recognized that water enclosed in a hydrophobic cleft could also be considerably lower in entropy than water at the surface of a convex hydrophobic solute. Consequently, its displacement can make a particularly favorable entropic contribution to binding.^[^ ^7^ ^]^ In general, binding site water whose displacement favors binding, whether enthalpically, entropically, or both, has been termed “high free energy”, “free energetically unfavorable”,^[^ ^8^ ^]^ “activated”,^[^ ^9^, ^10^ ^]^ “frustrated”,^[^ ^11^ ^]^ “unstable”, “xenophobic”, “unhappy”, or simply “unfavorable”.^[^ ^12^ ^]^

The role of water displacement as a determinant of binding thermodynamics has been extensively studied in supramolecular chemistry, well beyond the cyclodextrin and cyclophane macrocycles where it originated.^[^ ^13^, ^14^, ^15^, ^16^, ^17^, ^18^, ^19^, ^20^, ^21^ ^]^ For cucurbiturils, which stand out due to their ultrahigh affinity binding to hydrophobic guests,^[^ ^22^, ^23^, ^24^, ^25^ ^]^ the importance of thermodynamically unfavorable cavity water became apparent early^[^ ^26^, ^27^ ^]^ and stimulated the first computational descriptions.^[^ ^28^, ^29^, ^30^ ^]^ In addition, thermodynamic analysis of binding site water is nowadays widely used in structure‐based drug design,^[^ ^31^, ^32^, ^33^, ^34^, ^35^, ^36^, ^37^ ^]^ based on the expectation that ligands can gain affinity by displacing the water from protein subpockets where it is particularly unfavorable.^[^ ^34^, ^35^, ^38^ ^]^

Theoretical work on thermodynamic densities in fluids provides the foundation of these concepts,^[^ ^12^, ^39^ ^]^ and they are well connected to the results of atomistic simulations.^[^ ^7^, ^35^, ^36^, ^40^, ^41^, ^42^, ^43^, ^44^, ^45^, ^46^ ^]^ Nevertheless, the idea that the displacement of cavity water contributes to binding has been challenged, and critics have argued that the chemical potential of water in a solution is uniform and not position‐dependent, meaning there is nothing thermodynamically unique about water in a specific location, such as a binding cavity.^[^ ^47^, ^48^, ^49^ ^]^ This perspective, though, remains a topic of theoretical debate.^[^ ^12^ ^]^ Dewetting, or drying, of cavities has been offered as an alternative viewpoint,^[^ ^50^, ^51^ ^]^ but whether and how the “dried” nature of a cavity can drive binding remains unclear. In addition, a dried cavity is arguably an extreme manifestation of high‐energy water,^[^ ^52^, ^53^ ^]^ and it should be possible to bring the two concepts into accordance.

Here, we elucidate the thermodynamic contributions of water displacement through computational analysis of idealized, aqueous host‐guest systems specifically designed to address the issues raised above. We use molecular dynamics (MD) simulations to compute the binding free energy of a simple guest to a series of hosts that interact more or less favorably with water. At one extreme, water occupancy is so unfavorable that the binding site has dewetted. At the other extreme, water is tightly bound within a highly ionic host. We isolate the contributions of the solvent to binding by using a hard sphere that has negligible interactions with both the hosts and the solvent. This can be viewed as modeling a highly hydrophobic guest molecule. In addition, the series of host molecules includes an essentially “hard” host so that we can compute the affinity of the host‐guest system with near‐zero host‐guest, host‐solvent, and guest‐solvent interaction energies.

## Results and Discussion

### Model Systems and Calculations

**Figure 1:**
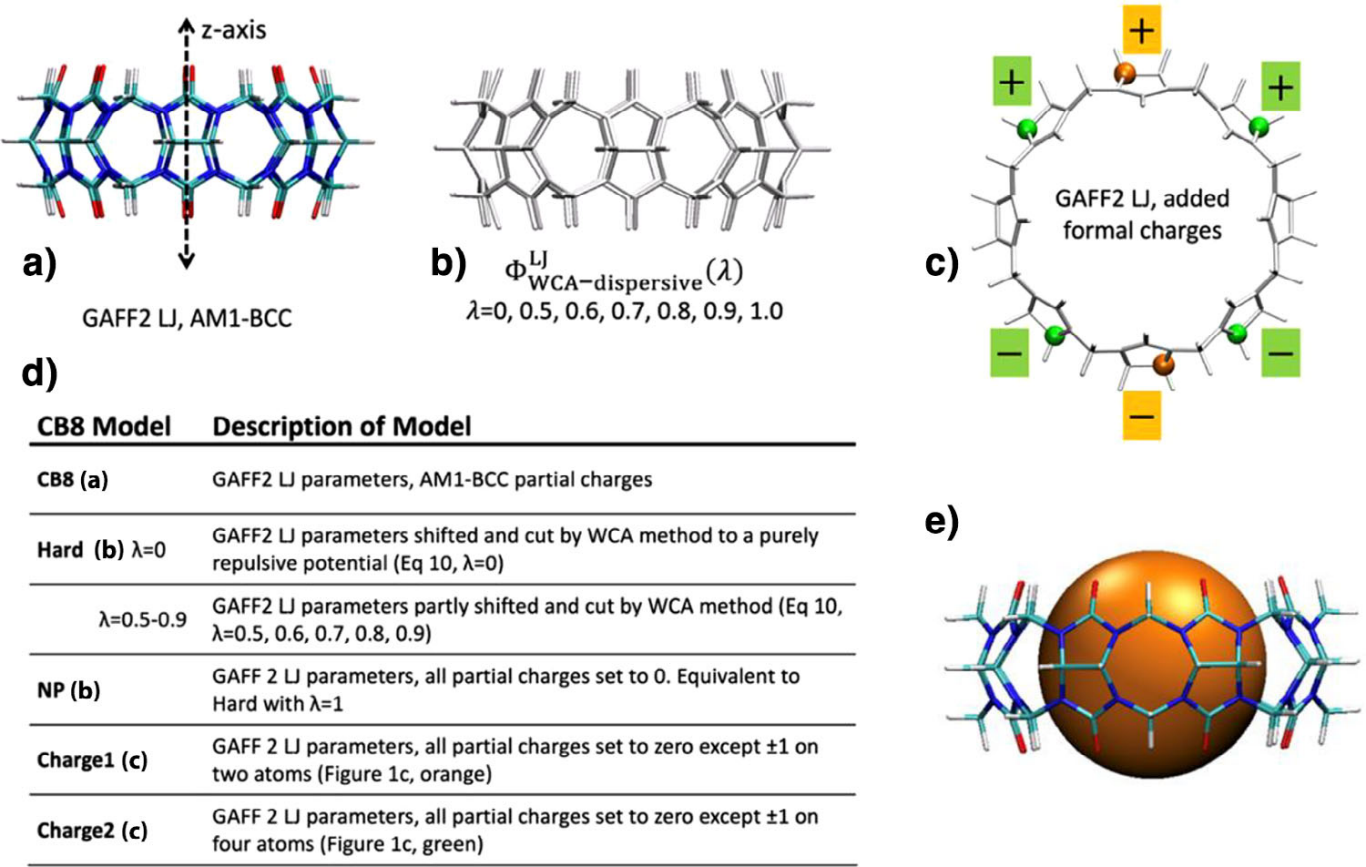
Models of the cucurbit[8]uril (CB8) host molecule studied here. All are treated as rigid. a) CB8 with full GAFF2 Lennard Jones (LJ) parameters and AM1‐BCC partial charges. The axis of symmetry (*z*‐axis) is labeled. b) No partial charges, LJ dispersive interactions scaled from λ  =  0 (Hard, no dispersion interactions) to λ = 1 (NP, full LJ), see Supporting Information, and c) no partial charges except for ± 1*e* placed on one pair (orange) or two pairs (green) of equatorial carbon atoms. d) Table summarizing the model hosts and their names. e) Schematic representation of CB8 with the pseudo‐hard sphere “guest” (orange) displacing all water molecules from the binding cavity.

We used MD simulations of idealized host‐guest systems in explicit water to isolate and study the contribution of water to binding thermodynamics; see Supporting Information for Methods. We chose a simple spherical guest that can be encapsulated inside macrocyclic hosts based on the structure of cucurbit[8]uril (CB8), for which the inner‐cavity (de)hydration effects are known to play an essential role (Figure 1). Any thermodynamic contribution to binding attributable to direct host‐guest interactions is eliminated by using a pseudo‐hard‐sphere potential (PHSP) for the guest; this hard sphere has no attractive component. We also treat the host and guest as rigid, i.e., without internal degrees of freedom, to avoid thermodynamic contributions to the binding free energies that result from conformational deformations and fluctuations.

In addition, instead of computing the standard free energy of binding, which accounts for entropy changes due to changes in the distribution of the relative coordinates of the two molecules on binding,^[^ ^54^ ^]^ we compute the change in free energy (i.e., the reversible work) of moving the guest from a fixed position outside the host to a fixed position in the host along its axis of symmetry (*z*‐axis, Figure 1a). The free energy as a function of *z* is also known as the potential of mean force (PMF). The size of the spherical guest is set to be small enough that it does not clash with the host when moved along the *z*‐axis. Therefore, the PMF is flat if there is no solvent‐mediated interaction between the host and guest, such as if the calculation were done in vacuo. If displacing solvent from the two molecules (i.e., host and guest) upon binding is favorable, then the PMF will fall as the guest enters the host. Conversely, if displacing the solvent is net unfavorable, the PMF will rise. Thus, the PMF provides a clean report of the contributions of solvent to the free energy of each host‐guest association.

The guest in all PMF computations is a single, spherical “atom” of radius 5.0 Å, which interacts with both the host and the water molecules with a PHSP, which has no attractive component and has a steeply rising repulsive component that approximates a hard‐sphere potential but can be accommodated in an MD simulation. In detail, the diameter of the hard sphere (10.0 Å) was chosen to fall marginally below the diametral atom‐to‐atom O─O distances of the carbonyl portals (10.1 Å in the CB8 structures used for the MD simulations), but above the widest equatorial van‐der‐Waals wall‐to‐wall C─C distances (ca. 9.8 Å from the CB8 crystal structure).^[^ ^55^ ^]^ It is important to note that the hard‐sphere potential was defined to only experience a sudden repulsion when an atomic nucleus would clash with the sphere. The selected diameter of the hard sphere ensures that it can be moved without clashes from outside the cavity into the center of the host in the PMF simulations and guarantees that there is no space for any residual water molecule inside the cavity of the host‐sphere complex. Consequently, all water molecules are displaced from the binding site of CB8 in the final host‐guest complex (Figure 1e).

As summarized in Figure 1, all hosts studied here share the structure of CB8. All host atoms are treated as fixed in position and interact with the guest via a PHSP. The baseline CB8 model interacts with water via a standard force field potential comprising Lennard‐Jones interactions and partial charges (Figure 1a). Additional CB8 models were created to explore the consequences of either reduced or strengthened attractive host interactions with water. First, to create a series of nonpolar CB8 models that interact more weakly with water, we set all partial charges to zero and introduced a coupling parameter λ (Figure 1b) which scales the strength of the host's dispersion interactions with water between an entirely non‐attractive host (Hard) at λ = 0 and an attractive but nonpolar host at λ = 1 (NP, see Supporting Information). Second, to investigate CB8 models with strongly favorable electrostatic interactions with water molecules, we created capacitor‐like constructs. Starting with the non‐polar NP model, charges of ± 1*e* were placed on one (Charge1) or two (Charge2) pairs of carbon atoms symmetrically, on opposite sides of the molecule (Figure 1c). All host models and the underlying interaction parameters are summarized in Figure 1d. Note that we selected CB8 as the base structure because this even‐membered macrocycle –unlike CB7–allows for the symmetric placement of opposite charges across from each other.

**Figure 2:**
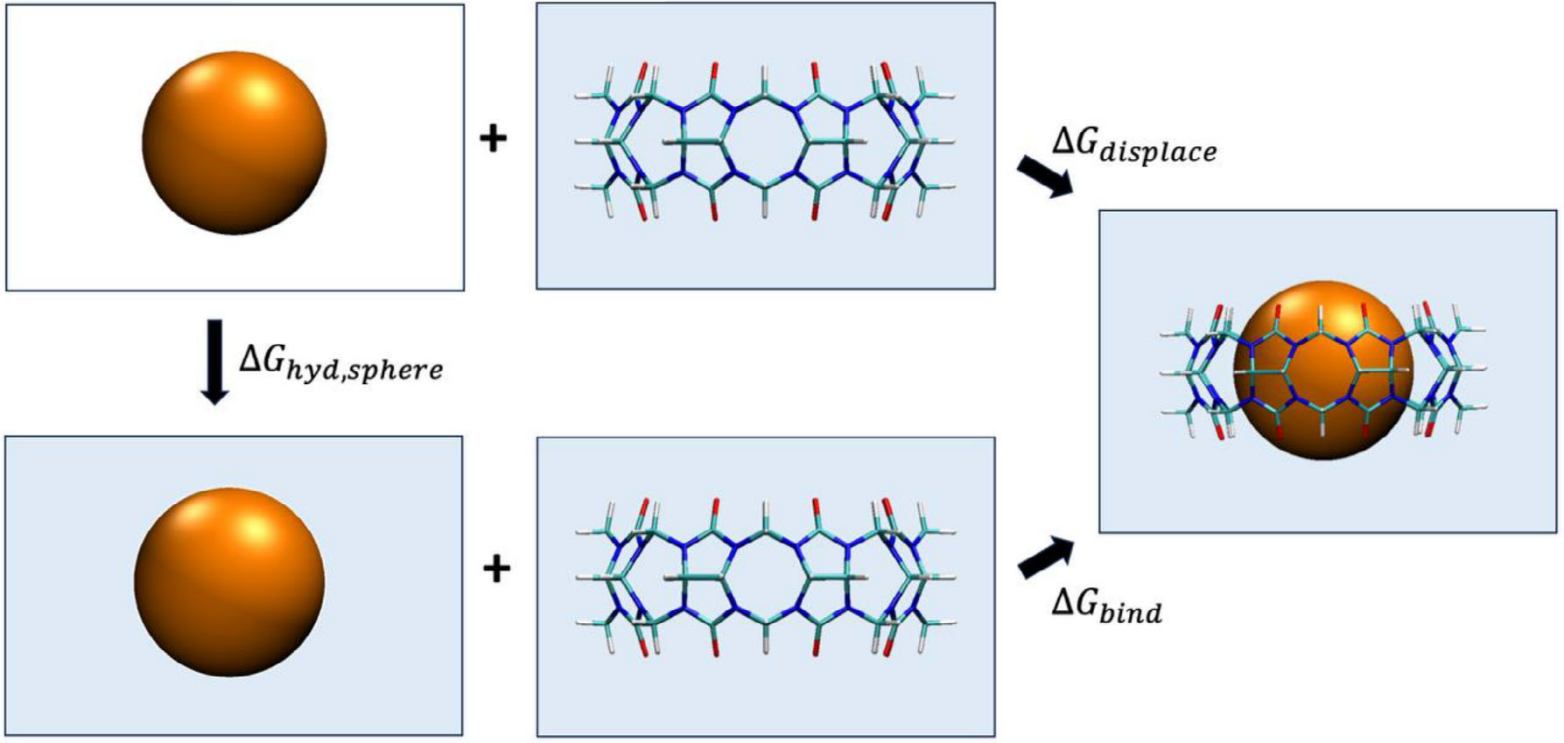
Thermodynamic cycle used to compute water displacement free energy. The lower process depicts binding of the PHSP guest initially in water (orange sphere, lower left) with water‐filled CB8 to form a bound complex (right). The top process shows binding of the PHSP guest initially in vacuum with the initially water‐filled host. The left process depicts the transfer of the PHSP guest from vacuum to water.

We employed a thermodynamic cycle (Figure 2) to determine the free energy change of fully displacing water from the binding site of the aqueous CB8 host, i.e., of forming a “bubble” in the binding site by inserting our PHSP guest. This quantity, Δ*G*_displace_, is given by the free energy change of moving the spherical guest, or bubble, from vacuum to water (i.e., of hydrating the guest) plus the free energy change of moving the guest into the binding site, i.e.: Δ *G*_displace_ =  Δ*G*_hyd,sphere_ + Δ*G*_bind_, where Δ*G*_bind_ is the change in the free energy when the sphere is moved from a fixed location in bulk water to the center of the CB8 cavity.

**Figure 3:**
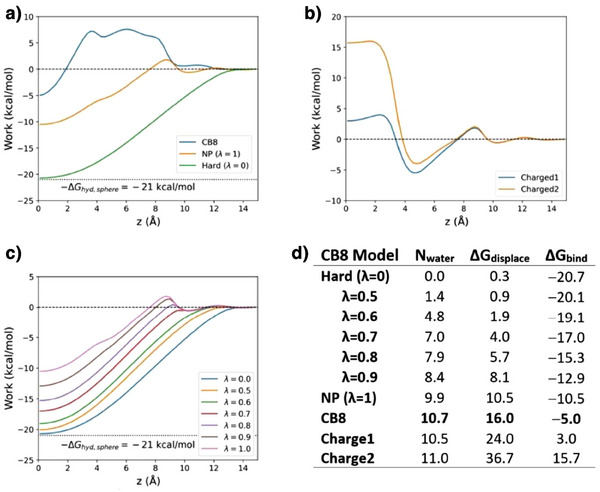
Computed thermodynamics of host‐guest binding for model systems. a) PMFs of pushing water molecules out of the CB8 cavity using the PHSP particle for CB8 (standard force field), NP, and Hard host models. b) PMFs for the charged models Charge1 and Charge2, c) PMFs for Hard model CB8 with intermediate strength dispersion interactions (*λ* = 0 to 1). d) Table showing, for each host considered in a)‐c), the mean number of water molecules in the binding cavity, N_water_, the binding free energy, and the water displacement free energy, all energies in kcal mol^−1^ (1 kcal mol^−1^ = 4.184 kJ mol^−1^). The number of water molecules in the cavity is estimated for a cylindrical region centered on the host with a radius 6.0 Å and height of ± 3.2 Å from the center of mass of CB8 along the *z*‐axis (see Figure 1a).

As shown in Figure 3a,d, the change in free energy upon moving the sphere from bulk water into the native CB8 binding site is Δ*G*_bind_ = −5 kcal mol^−1^. From simulations on the transfer of the hard sphere into aqueous solution, its hydration free energy (Δ*G*_hyd,sphere_) can be estimated as 21 kcal mol^−1^ with the same MD methods (see Supporting Information), which qualifies the hard sphere as highly hydrophobic, exaggerating the hydrophobicity of prototypal high‐affinity binders to cucurbiturils such as diamantane.^[^ ^23^, ^56^ ^]^ By using the thermodynamic cycle in Figure 2, we find a large free energy cost of +16 kcal mol^−1^ for displacing the binding site water. This result is consistent with the fact that the binding site is initially stably filled with 10.7 water molecules, on average, while this number falls to zero when the guest is bound. We then used the same method to determine the free energy of displacing water from the NP version of the CB8 host, where all atomic partial charges have been set to zero, and from the highly polar Charge1 and Charge2 versions of CB8, where all partial charges were zeroed and then either one pair (Charge1) or two pairs (Charge2) of atoms were assigned ± 1*e* charges to create zwitterionic hosts molecules (Figure 1).

The binding free energy of the PHSP guest with the NP host is considerably more favorable than for regular CB8, at Δ*G*_bind_ = −10.5 kcal mol^−1^ instead of −5.0 kcal mol^−1^ (Figure 3d). Using our thermodynamic cycle (Figure 2), the free energy cost of displacing the 9.9 water molecules present in NP is +10.5 kcal mol^−1^, much lower than the water displacement cost of +16.0 kcal mol obtained for CB8. We conclude that the water in the NP host is less thermodynamically favorable than the water in CB8 with its full complement of partial charges. This makes sense because, unlike native CB8, the fully nonpolar host cannot make favorable electrostatic interactions with the cavity water.

In contrast, the binding free energies of the PHSP guest with the highly polar Charge1 and Charge2 models of the CB8 macrocycle are unfavorable, at +3.0 kcal mol^−1^ and +15.7 kcal mol^−1^, respectively (Figure 3). This is because, as determined from the same thermodynamic cycle, the costs of water displacement have increased sharply to 24.0 kcal mol^−1^ and 36.7 kcal mol^−1^, respectively. However, the mean number of water molecules initially in the binding site is essentially unchanged, at 10.5 and 11.0, respectively. We conclude that the water in these highly polar hosts is much more thermodynamically favorable than the water in native CB8, due to the strong electrostatic interactions between the cavity water and the ionic charges of the hosts.

Increasingly dewetted binding cavities, with mean water occupancies approaching zero, can be obtained by not only setting the partial charges of the model CB8 host to zero but also scaling its Lennard‐Jones interactions with water from full LJ interactions (λ = 1, corresponding to host NP) down to a pseudo‐hard potential with no attractive component for λ = 0 (corresponding to host Hard). The mean number of water molecules in the cavity decreases steadily with decreasing lambda, with λ = 0 leading to essentially complete drying (Figure 3). Concurrently, the binding free energy becomes increasingly favorable, reaching −21 kcal mol^−1^ for the fully dewetted λ = 0 case. At the same time, the free energy cost of displacing water molecules drops to zero, consistent with the absence of water molecules to displace from the fully dried cavity, whose extremely low water occupancy is essentially that of water vapor under ambient conditions.

The free energy cost of displacing water from a binding cavity is greater than or equal to zero across all model hosts studied here. In this sense, water is stably present in the binding cavities of these hosts. However, the degree of stability varies, with the cost of water displacement ranging from 0 for the fully dewetted Hard host to +37 kcal mol^−1^ for the strongly polar Charge2 host. Because the free energy of dehydrating the spherical guest is the same across all of the macrocyclic hosts, and the interaction energy of all hosts with the guest is near zero, due to the pseudo‐hard‐sphere character of the guest, the initial thermodynamic state of the cavity water is the key determinant of affinity across this series of chemically different macrocycles. Accordingly, the free energies of binding range from −21 kcal mol^−1^ for the Hard host, where there is no water to be displaced, to +16 kcal mol^−1^ for Charge2, where the binding site water is tightly bound to the ionic host.

These results support the concept that the ability of a host molecule to achieve high affinity to a hydrophobic guest (modeled here by the hard sphere) depends on the presence of “unfavorable” binding site water. Cucurbiturils, for example, are known for their high binding affinities, attributed to their binding sites containing particularly unfavorable water. This is due to their barrel shape and nonpolar interiors,^[^ ^57^ ^]^ which deprive water molecules of the favorable interactions they typically have in bulk. In contrast, water in the binding sites of more open, polar, or aromatic hosts–such as cyclodextrins, calixarenes, and cyclophanes–is expected to be more thermodynamically favored, making its displacement less contributory to binding.^[^ ^14^ ^]^ It should be noted that the present calculations artificially isolate changes in water thermodynamics as the primary driver of binding, since the spherical guest is designed to have essentially zero direct interaction with the hosts. Experimentally, attractive forces between the guest and the host also contribute to binding and must be optimized to maximize affinity. However, the present results show that it is possible to achieve high binding affinities even without *any* optimization of attractive forces between host and guest.

**Figure 4:**
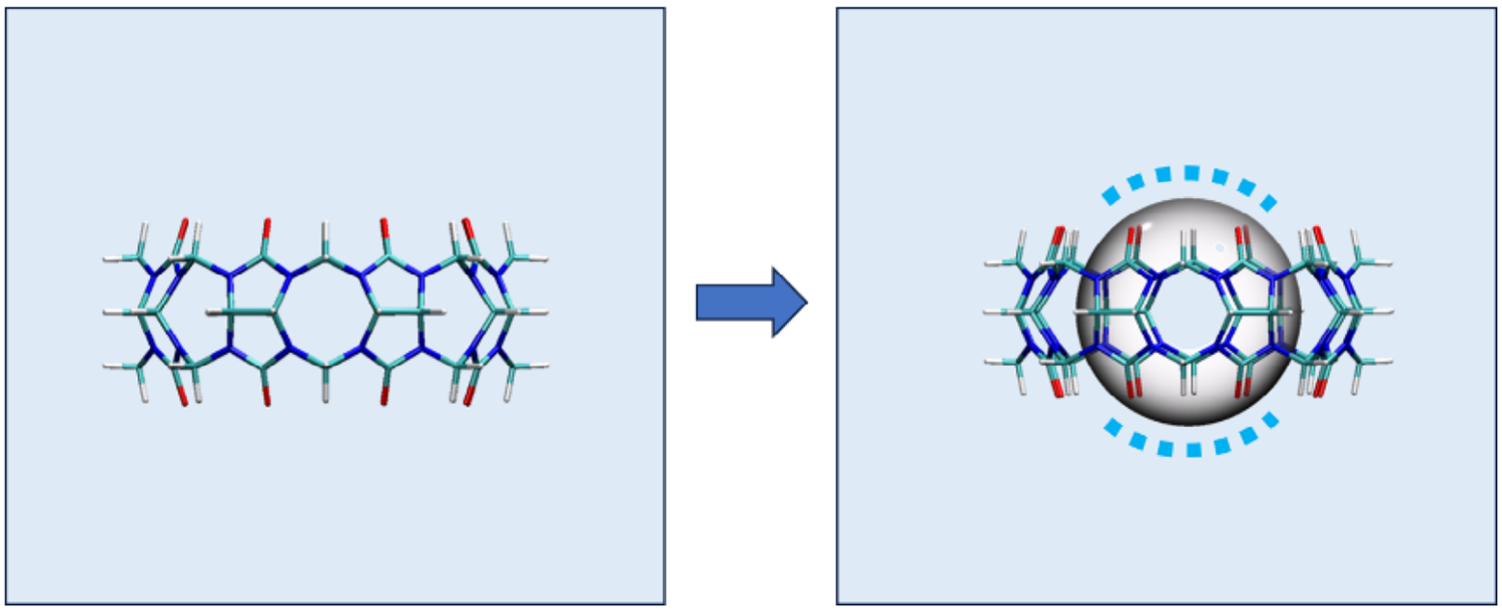
Hypothetical spontaneous dewetting of the CB8 host generates a bubble within the binding cavity. The dashed blue lines indicate thermodynamically unfavorable water at the surface of the bubble in the portal regions of CB8.

It may seem paradoxical that thermodynamically unfavorable water can be stably present in a binding site, such as in the NP host. One could argue along the same lines that–if the water inside a cavity is of “high free energy”–it would have no reason to remain there. However, spontaneous dewetting of our model CB8 binding sites does not occur and, hence, is not thermodynamically favored until virtually all attractive interactions with water are artificially eliminated, as in the Hard host. This may seem surprising, as the transfer of water from the cavity to the bulk would allow the thermodynamically unfavorable cavity water to adopt the more energetically favorable properties of bulk water. This paradox is resolved by recognizing that dewetting would create a new vapor‐liquid interface at the boundary of the dewetted region and that the liquid water at these interfaces (dashed lines in Figure 4) would itself be thermodynamically unfavorable. Therefore, when evaluating the stability of water in a binding site, it is crucial to consider both the initial and proposed final states.

**Figure 5:**
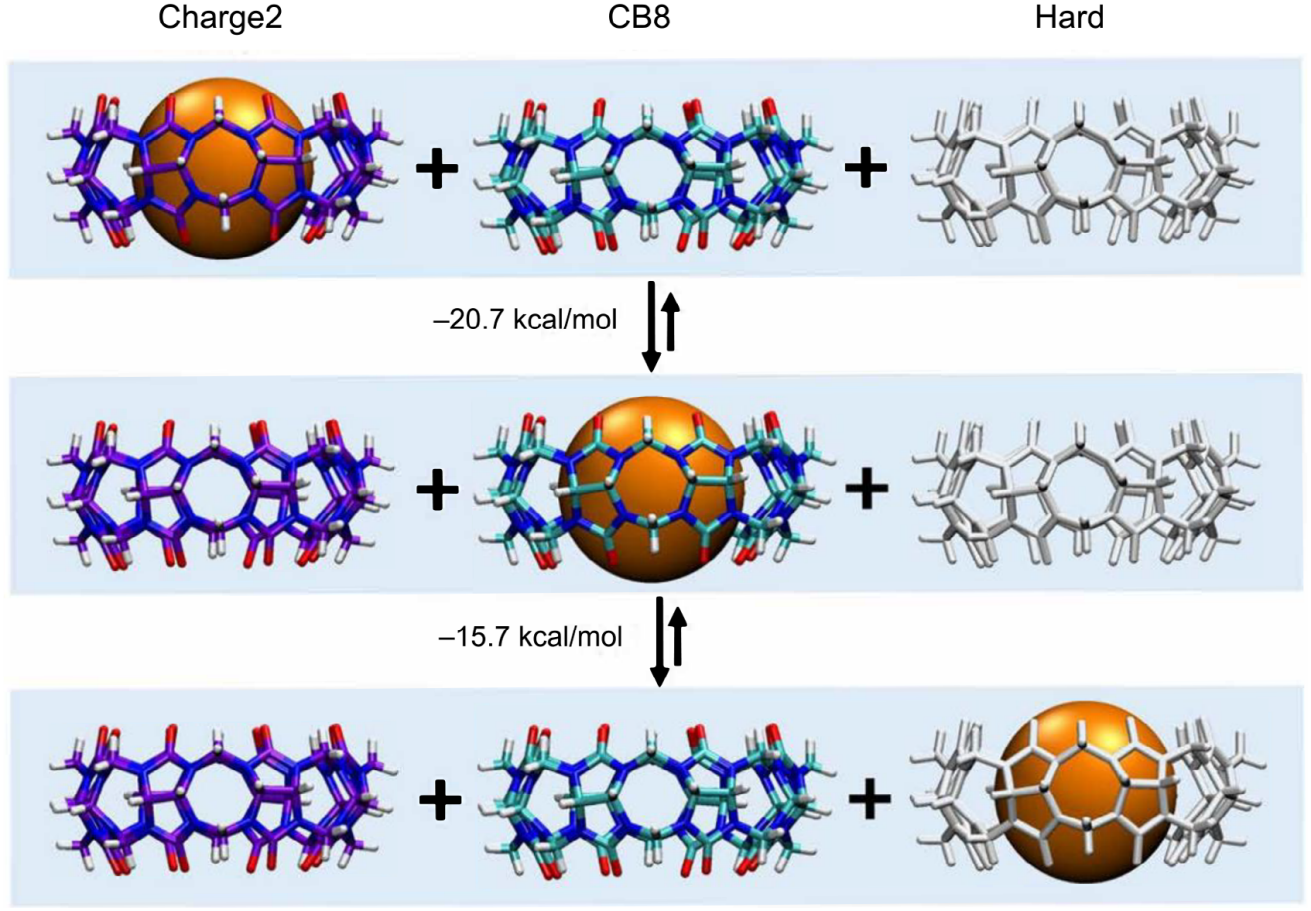
“Isodesmic” reactions highlight the contribution of water thermodynamics to binding. In water, the hard‐sphere guest (orange, not drawn to scale) binds most weakly to the Charge2 model, where cavity water is most stable, and most strongly to the Hard host model, where cavity water is least stable. The free energies are based on the results from Figure 3. In the gas phase, the hard‐sphere guest has no affinity to either host.

Given that the native CB8 cavity is stably solvated, it may be tempting to conclude that its thermodynamically favorable binding reaction is driven instead by the desolvation of the nonpolar guest. However, spontaneous dewetting of the guest is also thermodynamically disfavored, or else it would be surrounded by a vapor bubble. Thus, we have a situation in which binding could appear to be driven by two dehydration processes that are both unfavorable: that of the guest and that of the cavity of the host. Correct conclusions are reached only when we take a holistic view of the contribution of water to binding by considering the amount of unfavorable versus favorable water present in the initial versus final states of the system, instead of hypothetical dewetted or dehydrated states that do not arise in the actual system. The theory of the free energy density of fluids provides a rigorous foundation for this form of analysis.^[^ ^12^ ^]^ Accordingly, an informative way to focus on the role of water in binding is illustrated in Figure 5, where two “isodesmic” reactions are driven by the fact that water strongly prefers to be within the Charge2 binding site rather than within the binding site of native CB8, and that there is no free energy penalty for displacement of water from the Hard host on binding as it is already dewetted. Note that, if these reactions were to occur in vacuum, rather than in water, their free energy changes would be zero. The selectivity for binding the hard sphere to the different hosts in water is therefore very high and follows the order Hard >> CB8 >> Charge2.

It has been suggested that “water's influence on the free energy driving force for any aqueous self‐assembly necessarily depends entirely on the direct interactions between the solute species with each other and with water, rather than on indirect solute‐induced changes in water‐water interaction energy and entropy”,^[^ ^49^ ^]^ and a similar view has been expressed elsewhere.^[^ ^58^ ^]^ However, we observe strong binding of the PHSP guest with the Hard model host (Δ*G*_bind_ = −20.7 kcal mol^−1^), even though the energetic host‐guest, host‐water, and guest‐water interactions are weak and purely repulsive, and the changes in internal energy and configurational entropy of the host and guest are zero, since these idealized solutes have no internal degrees of freedom. Therefore, the strongly favorable binding free energy results purely from changes of water upon binding. Intuitively, binding is thermodynamically favored because it allows the free energy of the solvent –the volume integral of its free energy density^[^ ^12^ ^]^– to fall.

**Figure 6:**
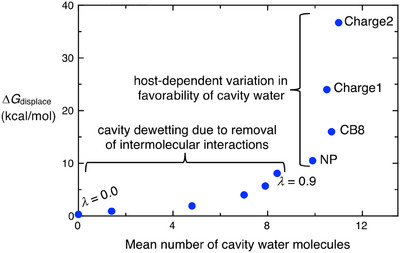
Relationship between the free energy of water displacement, Δ*G*_displace_ (kcal mol^−1^) and the mean number of cavity water molecules, for each model host‐guest system. Data are drawn from Figure 3d. Curly brackets highlight the biphasic character of the plotted relationship. For a plot on a logarithmic *x*‐axis, see Figure S5 in the Supporting Information.

Although the free energy costs of displacing water from the NP (λ = 1), CB8, Charge1, and Charge2 hosts range over 26 kcal mol^−1^, the mean number of cavity water molecules they contain is essentially constant, at 10.5 ± 0.6 (Figure 3d and Figure 6), which fully aligns with the expected number of water molecules inside the CB8 cavity.^[^ ^9^, ^11^, ^27^, ^28^ ^]^ The insensitivity of *N*_water_ to the strength of the attractive forces in this regime results from liquid water's low compressibility.^[^ ^59^ ^]^ In contrast, the entirely artificial host constructs with partial charges set to zero and λ < 1 show progressive dewetting as λ falls until finally *N*_water_ = 0 for λ = 0 (Hard model). Consistent with the weakened attractive forces between the host and the water and the falling number of water molecules to be displaced, the work of water displacement also falls, reaching a limiting value of 0 for the Hard model. However, these displacement free energies span only about 10 kcal mol^−1^. Thus, although this dewetting is an interesting phenomenon, we observe a larger thermodynamic variation among the host models that are fully hydrated (with liquid water). This demonstrates that, in the most common and experimentally most relevant setting of well‐hydrated binding cavities, water displaceability can vary widely without dewetting and correlates poorly, if at all, with water density. Although complete drying of binding sites may occur in small, highly nonpolar cavities and tubes, including the smaller cucurbit[5]uril homologue,^[^ ^14^, ^52^, ^53^ ^]^ it remains the exception to the rule that there is no vacuum in water, and most attention should be given to the more practically relevant situation of fully solvated hosts and guests.

As illustrated in Figure 4, the geometry of the CB8 cavity does not allow a full dehydration of the sphere due to the partially solvent‐accessible portal regions. Clearly, one would expect the thermodynamic consequences of dehydration to be less important for more “open” macrocyclic structures (such as cyclodextrins and calixarenes) and more pronounced for more confined supramolecular constructs (such as capsules or hemicarcerands).^[^ ^14^, ^60^ ^]^ The flexibility of a host may also play a role, as a host which is able to adapt to a guest better could lead to more complete guest desolvation, while the host prior to guest binding could contract its cavity and thereby minimize occupancy with unfavorable water.^[^ ^14^ ^]^ However, among macrocyclic hosts, cucurbiturils stand out due to the nonpolar and weakly polarizable nature of their inner cavity, their rigid structure, and associated high degree of guest confinement, which jointly leads to the presence of unfavorable encapsulated water molecules and which accounts, eventually, for their exceptionally high binding affinity.

As an additional key result, we observe a biphasic relationship between water density and the free energy of displacement (Figure 6). Thus, progressively eliminating all attractive host‐water interactions leads to progressive dewetting or drying of the cavity (lower left) but only modest changes in the free energy of water displacement, whereas progressively strengthening of attractive host‐water interactions causes no significant change in water density but dramatically increases the free energy cost of water displacement. This biphasic behavior could potentially be likened to a phase transition, though this interpretation remains speculative and warrants further detailed investigation to validate or refine this perspective.

The excess chemical potential of water at a given location, µ_*ex*_(*R*), is the free energy of inserting a water molecule at *R* so it might be expected to correlate with the thermodynamic favorability of water in our model host cavities.^[^ ^61^ ^]^ Because µ_*ex*_(*R*) varies linearly with ln ρ(*R*),^[^ ^61^, ^62^ ^]^ where ρ(*R*) is the number density of water, and because all of the present model hosts have cavities of identical volume, we can test this expectation by looking for a linear relationship between Δ*G*_displace_ and ln *N*_water_. From Figure 6, however, it is clear that this relationship does not hold, particularly among the more realistic water‐filled CB8 models on the right‐hand side of the graph. Thus, neither the density nor the excess chemical potential of water is a reliable indicator of the thermodynamic favorability of liquid water. As to the chemical potential of water, this quantity is fundamentally not a position‐dependent quantity. Rather, it is the change in free energy of adding one water molecule to the system, so it is influenced by all positions in the system that a water molecule accesses.

## Conclusion

The present computational analysis of simplified host‐guest systems clearly shows that binding affinities are strongly influenced by the thermodynamic properties of the water displaced from binding sites. These thermodynamic properties of water and the associated favorability of water displacement vary strongly with the chemical nature of the host. The results also demonstrate that significant cavity dewetting or even complete drying can be observed as a special, extreme case, for example, when polar interactions are removed and dispersion interactions are artificially reduced, and this effect can further promote host‐guest binding. Interestingly, neither water density nor excess chemical potential reliably indicates the thermodynamic favorability of cavity water. Although our hosts all share the structural framework of a cucurbituril, the concepts developed here are applicable to other macromolecular host molecules and, thus, can be broadly useful to explain observations and guide the design of pharmaceutical drugs and supramolecular systems.

## Supporting Information

The authors have cited additional references within the Supporting Information.^[^ ^63^, ^64^, ^65^, ^66^, ^67^, ^68^, ^69^, ^70^, ^71^, ^72^, ^73^, ^74^, ^75^, ^76^, ^77^, ^78^, ^79^ ^]^

## Conflict of Interests

MKG has an equity interest in and is a cofounder and scientific advisor of VeraChem LLC. He is also on the Scientific Advisory Boards of Denovicon Therapeutics, In Cerebro, Cold Start Therapeutics, and Beren Therapeutics.

## Supporting information



Supporting Information

## Data Availability

The data that support the findings of this study are available in the Supporting Information of this article.
