# Supplementary material for: Thermodynamics of Water Displacement from Binding Sites and its Contributions to Supramolecular and Biomolecular Affinity
Source: Angew Chem Int Ed Engl. 2025 Jun 16;64(35):e202505713. doi: 10.1002/anie.202505713 (PMC12377430; doi:10.1002/anie.202505713)
Supplement: Supplementary file 1 — Supporting Information [file ANIE-64-e202505713-s001.pdf]

Supporting Information for

On the Thermodynamics of Water Displacement from Binding Sites and its Contributions to Supramolecular and Biomolecular Affinity

Jeffry Setiadi,[a] Frank Biedermann,[b],* Werner M. Nau,[c],* Michael K. Gilson[a],*

[a] Dr. J Setiadi, Prof. Dr. M. K. Gilson
Skaggs School of Pharmacy and Pharmaceutical Sciences, 9255 Pharmacy Lane, University of California San Diego, La Jolla, CA, 92093, USA
E-mail: mgilson@ucsd.edu

[b] Priv.-Doz. Dr. F. Biedermann
Institute of Nanotechnology (INT), Karlsruhe Institute of Technology (KIT), Hermann-von-Helmholtz Platz 1, 76344 Eggenstein-Leopoldshafen, Germany
E-mail: frank.biedermann@kit.edu

[c] Prof. Dr. W. M. Nau
School of Science, Constructor University, Campus Ring 1, 28759 Bremen, Germany
E-mail: wnau@constructor.university

Computational Methods

Pseudo-Hard Potential Function of Guest. To focus on solvent contributions to binding free energy, we designed an extremely simple "guest molecule", i.e., one with a simple shape, no internal degrees of freedom, no attractive interactions with either the host or the water, and a repulsive potential that approximates a hard-wall potential, generating steric interactions while making only minimal contributions to the mean potential energy of the system. In particular, the steeper the rise of the repulsive potential with distance, the better. Accordingly, the guest is modeled as a single spherical "atom" of radius 5 Å, which is large enough to displace all water from the host cavity but small enough to pass through the carbonyl portals without generating steric clashes, and having an interaction potential that approximates the "hard wall" potential given by

$$\Phi_{\text{hard sphere}}(r_{ij}) = \begin{cases} \infty, & r_{ij} \leq \sigma \\ 0, & r_{ij} > \sigma \end{cases}, \tag{1}$$

where σ determines the particle size and r_{ij} is the interatomic distance. A true hard-wall potential cannot be used in a molecular dynamics (MD) simulation as the interatomic force is undefined at $r_{ij} = \sigma$. Therefore, we sought a differentiable approximation to $\Phi_{\text{hard sphere}}$, i.e., an intermolecular potential with no attractive component and a steeply increasing repulsive component.

However, designing a sufficiently "hard" potential for a 5 Å atom was not straightforward despite the popularity of hard-sphere models. We initially tried the cut-and-shifted Weeks-Chandler-Anderson (WCA) perturbation[1] of the Lennard-Jones (LJ) potential:

$$\Phi^{\text{LJ}}_{\text{WCA-repulsive}}(r_{ij}) = \begin{cases} \Phi_{\text{LJ}}(r_{ij}) + \epsilon, & r_{ij} \leq R^{\text{LJ}}_{\text{min}} \\ 0, & r_{ij} > R^{\text{LJ}}_{\text{min}} \end{cases}. \tag{2}$$

with

$$\Phi_{\text{LJ}}(r_{ij}) = 4\epsilon\left[\left(\frac{\sigma}{r_{ij}}\right)^{12} - \left(\frac{\sigma}{r_{ij}}\right)^{6}\right], \tag{3}$$

where ϵ is the depth of the LJ energy minimum located at

$$r_{ij} = R^{\text{LJ}}_{\text{min}} = \sigma 2^{\frac{1}{6}}. \tag{4}$$

However, simulations with this potential still yielded configurations with waters significantly closer than 5 Å from the center of the atom, leading to significantly positive mean interaction potentials. This problem results from the fact that increasing the value of σ softens the repulsive wall, as shown in the Figure S1A. Therefore, we substituted the more general Mie potential[2] for the LJ potential:

$$\Phi_{\text{Mie}}(r_{ij}) = \frac{c_r}{c_r - c_a}\left(\frac{c_r}{c_a}\right)^{\frac{c_a}{c_r - c_a}} \epsilon \left[\left(\frac{\sigma}{r_{ij}}\right)^{c_r} - \left(\frac{\sigma}{r_{ij}}\right)^{c_a}\right], \tag{5}$$

where c_r and c_a are the exponents of the repulsive and attractive terms, respectively. The minimum of the generalized Mie potential is positioned at

$$R^{\text{Mie}}_{\text{min}} = \sigma\left(\frac{c_r}{c_a}\right)^{\frac{1}{c_r - c_a}}, \tag{6}$$

and the WCA repulsive perturbation to the Mie potential is

$$\Phi^{\text{Mie}}_{\text{WCA-repulsive}}(r_{ij}) = \begin{cases} \Phi_{\text{Mie}}(r_{ij}) + \epsilon, & r_{ij} \leq R^{\text{Mie}}_{\text{min}} \\ 0, & r_{ij} > R^{\text{Mie}}_{\text{min}} \end{cases}. \tag{7}$$

Coefficients of $c_r = 50$ and $c_a = 49$ were chosen for the repulsive and attractive terms, respectively, following Jover et al.[2] However, for larger values of σ, the repulsive potential changes are, again, no longer as steep as desired (Figure S1B). To remedy this, we introduced a distance shift using a new variable, R_{particle}, which controls the particle size instead of σ,

$$r'_{ij} = r_{ij} - \left(R_{\text{particle}} - R_{\text{min}}^{\text{Mie}}\right), \tag{8}$$

yielding what appears to be a novel approximation to the hard-sphere potential for use in simulations:

$$\Phi_{\text{WCA-repulsive}}^{\text{Mie}}\left(r'_{ij}\right) = \begin{cases} \Phi_{\text{Mie}}\left(r'_{ij}\right) + \epsilon, & r_{ij} \le R_{\text{particle}} \\ 0, & r_{ij} > R_{\text{particle}} \end{cases}. \tag{9}$$

Values of 3 Å and 0.1 kcal/mol were used for σ and ϵ, respectively, and a particle radius R_{particle} of 5 Å. Note that, with this definition of the pseudo hard-sphere potential, the guest has essentially zero interaction with any atom whose center (nucleus) is more than 5 Å away, and has a strong steric clash with any atom whose center is less than 5 Å away.

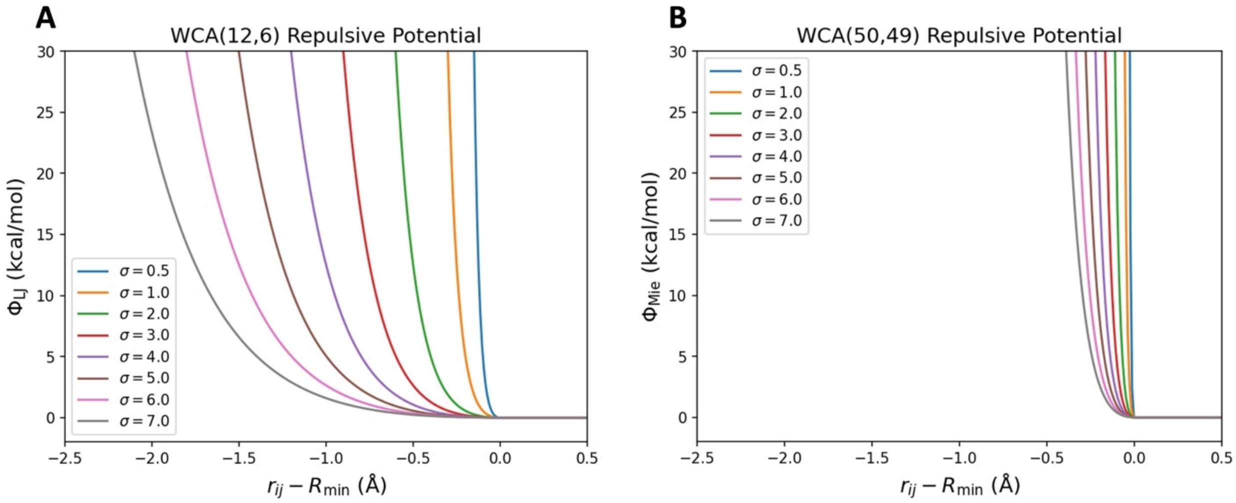

Figure S1. Graphs of candidate pseudo-hard sphere potentials plotted relative to the quantity $r_{ij} - R_{\text{min}}$ (shifted r_{ij}) to better superpose the curves for the cut-and-shifted WCA (A) 12-6 LJ potential and (B) 50-49 Mie potential. The σ values are reported in units of Å.

Hydration Free Energy of the Pseudo-Hard-Sphere Particle. We use physical (path-based) and alchemical free energy calculations to compute the hydration free energy (HFE), $\Delta G_{\text{hyd,sphere}}$, of our PHSP guest. In the alchemical calculations, we introduce a softcore potential function into Eq (5) to prevent the end-point catastrophe,[3] giving us an alchemically-modified, generalized Mie potential.[4]

$$\Phi_{\text{softcore}}^{\text{Mie}}\left(r_{ij}, \lambda\right) = \frac{c_r}{c_r - c_a}\left(\frac{c_r}{c_a}\right)^{\frac{c_a}{c_r - c_a}} \lambda\epsilon \left[\frac{1}{\left[\alpha(1-\lambda) + \left(\frac{r_{ij}}{\sigma}\right)^{c_a}\right]^{\frac{c_r}{c_a}}} - \frac{1}{\alpha(1-\lambda) + \left(\frac{r_{ij}}{\sigma}\right)^{c_a}}\right]. \tag{10}$$

Note that the location of the minimum varies with the coupling parameter λ:

$$R_{\text{min}}^{\text{Mie-softcore}}(\lambda) = \sigma\left[\left(\frac{c_r}{c_a}\right)^{\frac{c_a}{c_r - c_a}} - \alpha(1-\lambda)\right]^{\frac{1}{c_a}}. \tag{11}$$

Inserting the potential above in the cut-and-shifted WCA perturbation of Eq (9) gives

$$\Phi_{\text{WCA-repulsive}}^{\text{Mie-softcore}}(r'_{ij},\lambda) = \begin{cases} \Phi_{\text{Mie}}^{\text{softcore}}(r'_{ij},\lambda) + \lambda\epsilon, & r_{ij} \leq R_{\text{particle}} \\ 0, & r_{ij} > R_{\text{particle}} \end{cases}. \quad (12)$$

The alchemical calculations were performed over 15 windows (λ = 0.0, 0.05, 0.1, 0.15, 0.2, 0.25, 0.3, 0.35, 0.4, 0.5, 0.6, 0.7, 0.8, 0.9, 1.0). The simulation temperature and pressure were maintained at 298.15 K and 1 bar, respectively. For each window, we ran an energy minimization, followed by 1 ns of equilibration and 10 ns of production. The free energy was estimated using the Multistate Bennett-Acceptance-Ratio (MBAR) method[5].

As a numerical check of the alchemical method, we also computed the hydration free energy using a physical path-based method. To do this, we extended the periodic box vector in the z-axis by 40 Å, creating a vacuum region in the system, and simulated the system with the NVT ensemble. Water molecules were prevented from drifting with a flat-bottomed, harmonic wall restraint with a spring constant of 10 kcal/mol/Å^2, co-centered with the water-filled region and with its two walls positioned 5 Å above the upper (z large) and below (z small) the z boundaries of the initial water-filled region. Restraints of 1 kcal/mol/Å^2 were applied to the PHSP particle at intervals along the z-axis to define 31 umbrella sampling windows over a distance of 30 Å. We ran the same amount of sampling as in our alchemical calculations above, and the PMF was extracted with WHAM. The uncertainty in the PMF was estimated with 2000 steps of bootstrapping. The value of $\Delta G_{\text{hyd,sphere}}$ quantity was obtained as the difference between the PMF at z_i = 0 Å and 30 Å.

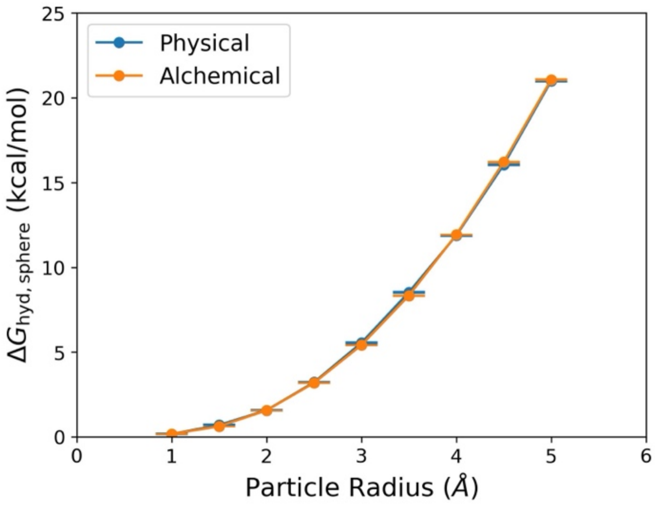

Figure S2. Comparison of the hydration free energy (HFE) of pseudo hard-sphere particles (PHSP) of various radii (R_{particle}), computed with the physical path-based and alchemical methods.

The physical and alchemical methods gave essentially the same value of $\Delta G_{\text{hyd,sphere}}$ as we varied the particle radius from 1 Å to 5 Å (Figure S2 and Table S1). The value of $\Delta G_{\text{hyd,sphere}}$ is positive for all radii, indicating a free energy cost of creating a spherical cavity, or bubble, in bulk water. The free energy cost increases sharply as the particle becomes larger, reaching a value of 21 kcal/mol at 5 Å, the size used in the subsequent binding (PMF) calculations. The present results, obtained with the TIP3P water model, are similar to those reported previously[6] for hydration of hard spheres of various radii with the TIP4P[7] water model. Because the PHSP potential has no attractive component and a steep-walled ("hard") repulsive component, the mean interaction potential energy of the hydrated 5 Å particle with water is only 0.30 kcal/mol – a very small value relative to the hydration free energy of 21 kcal/mol, as intended. The near absence of solute-solvent interactions implies that the strongly positive hydration free energy results almost entirely from the reorganization of the water in response to the insertion of the particle (cavitation energy). These results provide a methodological foundation for the present study.

Table S1. The change in the free energy of hydration of the pseudo-hard-sphere particle, computed with the physical and alchemical methods, for different particle sizes.

R_{particle} (Å)	$\Delta G_{\text{hyd,sphere}}^{\text{Physical}}$ (kcal/mol)	$\Delta G_{\text{hyd,sphere}}^{\text{Alchemical}}$ (kcal/mol)
1.0	0.17 ± 0.03	0.17 ± 0.00
1.5	0.70 ± 0.04	0.62 ± 0.00
2.0	1.57 ± 0.04	1.56 ± 0.01
2.5	3.22 ± 0.04	$3.18 + 0.01$
3.0	5.55 ± 0.05	5.41 ± 0.01
3.5	8.53 ± 0.04	8.33 ± 0.02
4.0	11.88 ± 0.04	11.92 ± 0.02
4.5	16.05 ± 0.04	16.21 ± 0.03
5.0	20.99 ± 0.04	21.07 ± 0.03

Models of the CB8 Host Molecule. All of the model hosts considered here (Figure 1 in main text) interact with the PHSP guest via Eqs (5)-(9), but we varied the character of the hosts' interactions with water. Our baseline CB8 model is assigned conventional force field nonbonded parameters (see below). To create a series of nonpolar CB8 models whose attractive LJ (dispersion) interactions with water range down to zero, we set all partial charges to zero and used the following equation to scale from full LJ interactions (NP) to the cut-and-shifted WCA potential (Hard):

$$\Phi_{\text{WCA-dispersive}}^{\text{LJ}}(r_{ij},\lambda) = \begin{cases} \Phi_{\text{LJ}}(r_{ij}) + \epsilon(1-\lambda), & r_{ij} \le R_{\min}^{\text{LJ}} \\ \lambda\Phi_{\text{LJ}}(r_{ij}), & r_{ij} > R_{\min}^{\text{LJ}} \end{cases}. \quad (13)$$

Here λ is a coupling parameter between 0 and 1 that determines the strength of the LJ dispersive interactions. Using this potential provides a non-attractive host (Hard) at λ = 0 and an attractive but nonpolar host (NP) at λ = 1. The mean potential energy of interaction of the Hard host with all water in the simulation system is small and unfavorable, at 2.8 kcal/mol, consistent with the design of the pseudo-hard potential. To create the capacitor-like constructs Charge1 or Charge2 (Figure 1C in main text) from the non-polar NP model, charges of $\pm 1e$ were placed on one or two pairs of carbon atoms symmetrically, on opposite sides of the molecule. To compute the mean number of waters in the different host cavities, we defined the cavity as a cylinder centered at the center of mass of the CB8 molecule and oriented along its axis of symmetry (*z*-axis, Figure 1A), with radius 6.0 Å and end-to-end length of 6.4 Å. The host molecule was treated as rigid in the present calculations in order to isolate the role of water in binding. In the geometry used, the distance between oxygen atoms diametrically across the portals averages 10.1 Å, just large enough to allow the hard guest with 5 Å radius to pass through without clash.

Potential of Mean Force Calculations. We used umbrella sampling (US) calculations with the weighted histogram analysis method (WHAM)[8] to compute potentials of mean force (PMF) for insertion of the PHSP guest into the host models along the axis of symmetry (z-axis) of the symmetric CB8 molecule (Figure S3). The reaction coordinate is the distance, projected onto the z-axis, between the center of the monatomic guest and the center of mass (COM) of CB8. We first pull the particle out of the CB8 host with steered MD (SMD) simulation, using a spring constant of 25 kcal/mol/Å^2, from 0 Å to 15 Å. A harmonic potential with a spring constant of 100 kcal/mol/Å^2 in the *x-y* plane was used to keep the particle close to the z-axis. The velocity was set to 2 Å/ns, and snapshots from these SMD simulations were used as the starting configuration for the subsequent US calculations. To improve the overlap between neighboring umbrella windows, we stratified the umbrella windows from 0 to 5 Å with increments of 0.25 Å and a spring constant of 25 kcal/mol/Å^2 and increments of 0.5 from 5 to 15 Å with a spring constant of 10 kcal/mol/Å^2. The same harmonic potential in the x-y plane was applied for each window as in the SMD calculations to limit the particle's motion away from the z-axis. The CB8 structure is kept rigid for all calculations to remove all internal, translational, and rotational degrees of freedom.

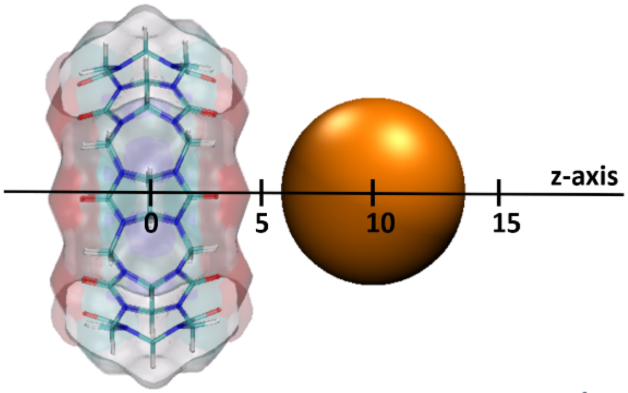

Figure S3. Diagram of PHSP guest (orange) at z = 10 Å while being inserted into the CB8 binding cavity (sticks and transparent surface).

Force Field and Simulation Details. For all CB8 model hosts, we used General Amber Force Field version 2.1 (GAFF2) LJ parameters. Note that the valence parameters are irrelevant because the molecules are treated as rigid. AM1-BCC[9,10] partial charges were generated with the *antechamber* program from AmberTools.[11] For the PMF calculations, we solvated the CB8 structure with 2500 TIP3P[7] water molecules positioned in the center of a rectangular periodic box with the *tleap* program of AmberTools as shown in Figure S4. For HFE calculations, we solvated the PHSP particle or CB8 with 2000 TIP3P water molecules in a cubic box. All MD simulations were carried out with the OpenMM[12] MD engine, version 7.5.1. The short-range, direct, nonbonded interactions were truncated with a cutoff of 9 Å, and the long-range interactions were handled with the particle-mesh Ewald (PME) method.[13,14] The simulations were run in the NPT ensemble, maintaining the temperature at 298.15 K with the Langevin thermostat[15] with an integration time step of 2 fs. The system was maintained at 1.0 atmosphere pressure with the Monte Carlo barostat.[16,17] We implemented the potential functions of Eqs (5)-(13) and (10)-(12) in OpenMM[12] using the *CustomNonbondedForce* class. All the Python code used to model the CB8 and PHSP systems and analysis used in this work is freely available on GitHub.[18]

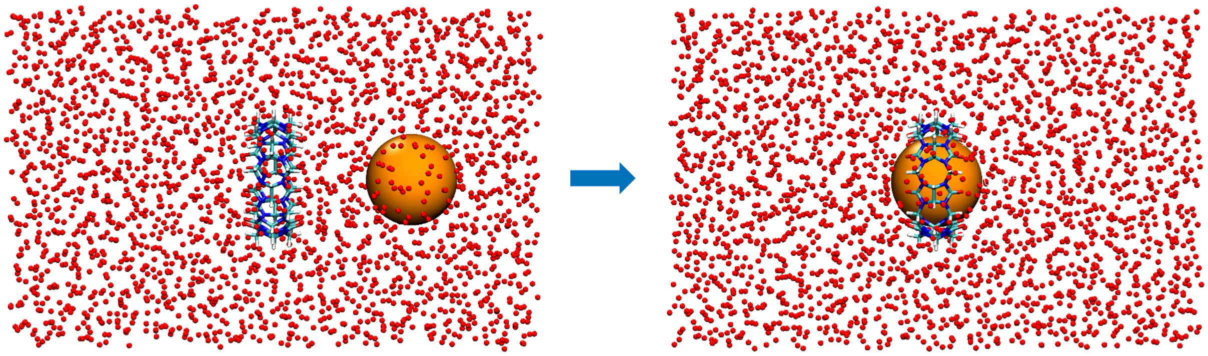

Figure S4. Snapshots of the host (sticks) and guest (orange sphere) with 2000 water molecules (oxygen atoms shown as small red spheres) in rectangular simulation boxes. Left: unbound state. Right: bound state.

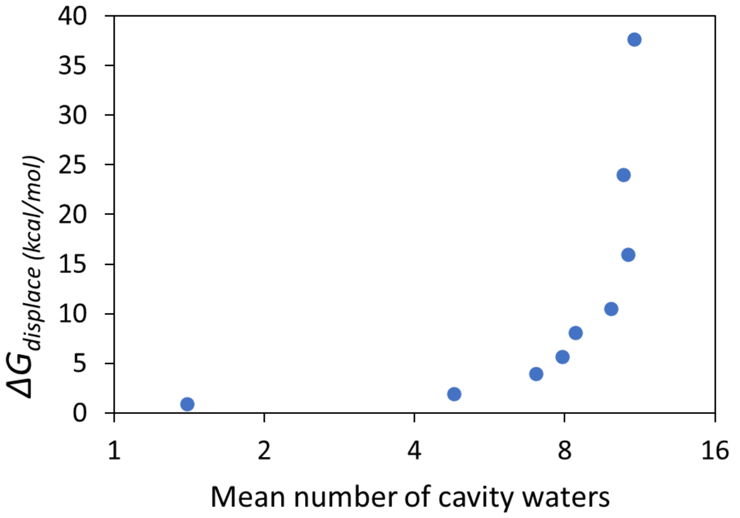

Figure S5. Free energy of water displacement as a function of the mean number of cavity waters, here plotted on a *logarithmic* horizontal axis. The data are the same as those plotted in Figure 6 in the main text.

References

[1] J. D. Weeks, D. Chandler, H. C. Andersen, *J. Chem. Phys.* **1971**, *54*, 5237-5247.

[2] J. Jover, A. J. Haslam, A. Galindo, G. Jackson, E. A. Müller, *J. Chem. Phys,* **2012**, *137*, 144505.

[3] T. C. Beutler, A. E. Mark, R. C. van Schaik, P. R. Gerber, W. F. van Gunsteren, *Chem. Phys. Lett.,* **1994,** *222,* 529-539.

[4] I. Q. Matos, C. R. A. Abreu, F*luid Phase Equilib.* **2019**, *484*, 88-97.

[5] M. R. Shirts, J. D. Chodera, *J. Chem. Phys,* **2008**, *129*, 124105.

[6] F. M. Floris, *J. Phys. Chem. B,* **2005**, *109,* 24061-24070.

[7] W. L. Jorgensen, J. Chandrasekhar, J. D. Madura, R. W. Impey, M. L. Klein, *J. Chem. Phys*, **1983**, *79*, 926-935.

[8] S. Kumar, J. M. Rosenberg, D. Bouzida, R. H. Swendsen, P. A. Kollman, *J. Comput. Chem.*, **1992**, *13*, 1011-1021.

[9] A. Jakalian, B. L. Bush, D. B. Jack, C. I. Bayly, *J. Comput. Chem.*, **2000**, *21*, 132-146.

[10] A. Jakalian, D. B. Jack, C. I. Bayly, *J. Comput. Chem.,* **2002**, *23*, 1623-1641.

[11] D. A. Case, H. M. Aktulga, K. Belfon, I. Ben-Shalom, S. R. Brozell, D. S. Cerutti, T. E. Cheatham III, V. W. D. Cruzeiro, T. A. Darden, R. E. Duke, *Amber 202*1, University of California, San Francisco, **2021**.

[12] P. Eastman, J. Swails, J. D. Chodera, R. T. McGibbon, Y. Zhao, K. A. Beauchamp, L.-P. Wang, A. C. Simmonett, M. P. Harrigan, C. D. Stern, R. P. Wiewiora, B. R. Brooks, V. S. Pande, *PLoS Comput. Biol.*, **2017**, *13*, e1005659.

[13] T. Darden, D. York, L. Pedersen, *J. Chem. Phys.*, **1993**, *98*, 10089-10092.

[14] U. Essmann, L. Perera, M. L. Berkowitz, T. Darden, H. Lee, L. G. Pedersen, *J. Chem. Phys.*, **1995**, *103*, 8577-8593.

[15] J. A. Izaguirre, C. R. Sweet, V. S. Pande, in *Biocomputing* **2010**, pp. 240-251.

[16] K.-H. Chow, D. M. Ferguson, Comput. *Phys. Commun.*, **1995**, *91*, 283-289.

[17] J. Åqvist, P. Wennerström, M. Nervall, S. Bjelic, B. O. Brandsdal, *Chem. Phys. Lett.*, **2004**, *384*, 288-294.

[18] GitHub, https://github.com/jeff231li/pseudo-hard-sphere-solvation-scripts, **2025**.
